# Characterization of CgHIFα-Like, a Novel bHLH-PAS Transcription Factor Family Member, and Its Role under Hypoxia Stress in the Pacific Oyster *Crassostrea gigas*

**DOI:** 10.1371/journal.pone.0166057

**Published:** 2016-11-04

**Authors:** Ting Wang, Jie Meng, Li Li, Guofan Zhang

**Affiliations:** 1 Key Laboratory of Experimental Marine Biology, Institute of Oceanology, Chinese Academy of Sciences, Qingdao, China; 2 University of Chinese Academy of Sciences, Beijing, China; 3 Laboratory for Marine Biology and Biotechnology, Qingdao National Laboratory for Marine Science and Technology, Qingdao, China; 4 Laboratory for Marine Fisheries and Aquaculture, Qingdao National Laboratory for Marine Science and Technology, Qingdao, Shandong, China; 5 National & Local Joint Engineering Laboratory of Ecological Mariculture, Institute of Oceanology, Chinese Academy of Sciences, Qingdao, China; University of Hawaii System, UNITED STATES

## Abstract

Hypoxia-inducible factor (HIF), a critical member of the basic-helix-loop-helix (bHLH)-containing Per-Arnt-Sim (PAS) protein family, is a master transcription factor involved in maintaining oxygen homeostasis. In the present study, we isolated and characterized a novel bHLH-PAS family member, *CgHIFα-like* gene, from the Pacific oyster *Crassostrea gigas*, and determined its importance during hypoxia stress. The 3020-bp *CgHIFα-like* cDNA encoded a protein of 888 amino acids. The predicted CgHIFα-like amino acid sequence was conserved in the N-terminal bHLH, PAS, and PAC domains (but not in the C-terminal domain) and was most closely related to the HIF family in the bHLH-PAS protein phylogenic tree. Similar to the mammalian *HIF-1α*, *CgHIFα-like* could be expressed as four mRNA isoforms containing alternative 5′-untranslated regions and different translation initiation codons. At the mRNA level, these isoforms were expressed in a tissue-specific manner and showed increased transcription to varying degrees under hypoxic conditions. Additionally, the western blot analysis demonstrated that CgHIFα-like was induced by hypoxia. Electrophoretic mobility shift assay indicated that CgHIFα-like could bind to the hypoxia responsive element (HRE), whereas dual-luciferase reporter analysis demonstrated that CgHIFα-like could transactivate the reporter gene containing the HREs. In addition to CgHIFα-like, we identified CgARNT from the *C*. *gigas*, analyzed its expression pattern, and confirmed its interaction with CgHIFα-like using a yeast two-hybrid assay. In conclusion, this is the first report on the cloning and characterization of a novel hypoxia transcription factor in mollusks, which could accumulate under hypoxia and regulate hypoxia related gene expression by binding to HRE and dimerizing with CgARNT. As only one member of HIF has been identified in invertebrates to date, our results provide new insights into the unique mechanisms of hypoxia tolerance in mollusks.

## Introduction

Oxygen is critical for energy generation by aerobic respiration, its deprivation being lethal for many animals. As most of the metazoan organisms cannot survive without oxygen, its homeostasis must be strictly regulated in these organisms. Marine bivalves, including the Pacific oyster *Crassostrea gigas*, face limited oxygen conditions in intertidal zones. During low tide, the oysters are periodically exposed to desiccation, potentially leading to hypoxia or anoxia [1, 2]. In addition, increasing water eutrophication and global warming could induce hypoxia for oysters in seawaters [3]. Unlike fish and plankton, oysters have movement disability; they are, therefore, more susceptible to such hypoxia stress [4–6]. However, their survival for up to 47.8 days under desiccated conditions at 4°C indicates that oysters have evolved powerful mechanisms for tolerance to varying degree of hypoxia [2, 7–10]. During moderate hypoxia, oysters first use physiological mechanisms to stay aerobic, and an increase in ventilation of respiratory surfaces (mainly gills) is a common response to maintain their rate of oxygen consumption. This mechanism remains functional until the hypoxia threshold is reached (around 2mg/L O_2_), the oxygen tension below which is referred to as “severe hypoxia” [11, 12]. Upon exposure to severe hypoxia, anaerobiosis commences and oysters present biochemical adaptions including (1) extensive consumption of substrates for energy provision during the beginning of severe hypoxia (mainly phosphagens, aspartate and glycogen), (2) optimized fermentation with higher ATP output and the formation of non-acidic or volatile end-products such as succinate, propionate, and acetate [13–15], (3) strong depression of metabolic rate [16, 17], and (4) high expression of selective hypoxia-related genes [18, 19].

The hypoxia-inducible factors (HIFs) play important roles in molecular adaption to hypoxia [20–22]; they function as transcription factors, triggering expression of several genes during adaptive and survival responses to oxygen deprivation. HIF is a heterodimeric DNA-binding complex, consisting of a unique oxygen-dependent α subunit (HIF-1α/HIF-2α/HIF-3α in mammals) [23] and a common constitutively expressed β subunit (also termed as the aryl hydrocarbon receptor nuclear translocator, ARNT). Most of our knowledge about this important member of the basic helix–loop–helix/Per–Arnt–Sim bHLH/PAS family is derived from studies on mammalian HIF-1α; it contains one characteristic bHLH domain, two PAS domains, one domain C-terminal to PAS motifs (PAC), one oxygen-dependent degradation (ODD) domain, and two transactivation domains (N-TAD and C-TAD) [24]. The biological activity of HIF-1 is primarily determined by the abundance and transcriptional activity of its α subunit, which are tightly regulated by the level of oxygen [24–27]. Under normoxia, HIF-1α is hydroxylated by prolyl-4-hydroxylase (PHD) [28–30], followed by its ubiquitination and degradation [31, 32]. However, under hypoxia, HIF-1α accumulates, heterodimerizes with ARNT, and subsequently induces transcription of hypoxia-inducible genes by binding to the hypoxia response element (HRE) on the promoter/enhancer regions [33, 34].

HIF-α is widely conserved across metazoans [35–38]. Generally, there are three *HIFα*, namely *HIF-1α*, *HIF-2α*, and *HIF-3α* in mammals and only a single-copy of *HIF-α* in invertebrates, as revealed by the analysis of 50 eukaryote genomes [39]. In invertebrates, insect HIF-α homologs have both the N- and C-terminal oxygen-dependent degradation domains (for example, the homologs from the honeybee *Apis mellifera*, grass shrimp *Palaemonetes pugio*, and the red flour beetle *Tribolium castaneum*) [40, 41]; the nematode *Caenorhabditis elegans* and the cnidarian *Nematostella vectensis* HIF-α have only C-terminal ODD [40–45], whereas, the Tablet animal (*Trichoplax adhaerens*) HIF-α shows large variation in the ODD domains [39]. However, the extrapolation of one HIF oxygen-sensing system onto other species should be performed with caution [35, 36, 44]. For example, hypoxia-tolerant cyprinid fishes appear to retain six *HIF-α* gene copies (referred to as *HIF-1A/B*, *HIF-2A/B*, and *HIF-3A/B*) [46]. Moreover, compared to other insects, fruit fly (*Drosophila melanogaster*), a model organism, has only one ODD motif [47]. Overall, the number of HIF-α isoforms, as well as the molecular complexity of HIF-α has increased over the course of metazoan evolution, but other factors like the variable environmental oxygen levels have also affected the rate and mode of HIF-α evolution.

*HIF-α* has been cloned and characterized in marine animals, namely Eastern oyster, nassariid gastropods (*Nassarius siquijorensis* and *Nassarius conoidalis*), small abalone *Haliotis diversicolor*, mussel *Mytilus galloprovincialis*, and the Pacific oyster *C*. *gigas* [1, 48–51]. Based on oyster genome search, we found two genes annotated as *HIF-α* orthologs. Kawabe et al. (2012) cloned one of the *HIF-α* and investigated its role in response to air exposure [1]; its role in respiratory burst has been further studied using RNAi [52]. However, to date, there has been no investigation on the other member, referred to as *HIFα-like* in the present study. To functionally analyze the novel *C*. *gigas* HIFα-like (CgHIFα-like) protein, we examined whether: (i) *CgHIFα-like* could be induced by hypoxia treatment both at the mRNA and protein levels, (ii) the CgHIFα-like gene product could interact with CgARNT, and (iii) CgHIFα-like protein was capable of binding to HRE and possessed HRE-dependent transcriptional activity.

## Materials and Methods

### Ethics Statement

The Pacific oysters, *C*. *gigas*, used in this study were marine-cultured animal and were cultured in the aquarium at the Institute of Oceanology, Chinese Academy of Sciences (IOCAS) Qingdao, China. All the experiments were conducted according to local and national regulations. No specific permissions were required for oyster sample collection and for the experiments described in thise study. All the field studies were carried out at IOCAS, and did not involve any endangered or protected species.

### Experimental Animals

The Pacific oysters (*C*. *gigas*) were collected from Qingdao, Shandong province, China. Animals were acclimated at 22 ± 1°C in tanks with aerated and filtered seawater for more than one week before the experiment. For the tissue-specific expression analysis of *CgHIFα-like*, three types of oxygen-sensitive tissues, namely adductor muscles, gills and hemolymph, were collected from 18 oysters. To determine the effects of reduced oxygen levels on the gene expression, we segregated the adult animals in two groups and transferred them to normoxic (dissolved oxygen (DO) level, 7.0–7.4 mg/L, 100% of the normal oxygen level) or hypoxic (1.2–1.4 mg/L DO, ~20% of the normal oxygen level) conditions at 22 ± 1°C. The hypoxic condition of water was achieved by bubbling nitrogen-oxygen gas mixture into the water during the experiment. Normoxic conditions were maintained by simply bubbling atmospheric air. Throughout each treatment, the pO_2_ was monitored and regulated using an oxygen electrode (YSI556MPS, YSI, USA).Gills were sampled from six healthy oysters after hypoxic treatment for 72h, and were pooled equally as one sample. These three pooled samples were obtained for RNA extraction and gene expression analyses.

### cDNA Cloning and Sequence Analysis

The cDNA sequence of *CgHIFα-like* was obtained by searching the Pacific oyster *C*. *gigas* genome database (http://www.oysterdb.com). According to the predicted coding sequence (CDS), we designed primers to amplify the middle fragment from the oyster cDNA template. The rapid amplification of 3′- and 5′-cDNA ends (RACE) was performed to obtain the full-length cDNA. Briefly, 3′-RACE was conducted using specific forward primers (CgHIFα-like F1, F2 and F3) and a universal primer Oligo (dT)-adaptor (Table 1), according to the manufacturer’s instructions (Invitrogen, Carlsbad, CA, USA), whereas the 5′-end was cloned using specific reverse primers (CgHIFα-like R1, R2 and R3) and Oligo (dG)-adaptor (Table 1) from the dCTP-tailed cDNA template (Invitrogen, Carlsbad, CA, USA).

**Table 1:** Primers used in this study.

Primer name	Primer sequence (5′-3′)
5′-RACE of CgHIFα-like	
R1	ATCCCGATTGCTTTGTTCTAAT
R2	CTAAGTATTGCTTCATCCGTAAATA
R3	TTTCTACCGCCTCTTTATCTCG
3′-RACE of CgHIFα-like	
F1	GACGGACTGATCTGCATTTGCC
F2	GAATGTCCAAATCGCTGCCCTTA
F3	GGGAACCACATTGCGGTGCCCAGTA
semi-quantitative PCR	
CgHIFαlike- qRT-F	AGGACTCGGAGCCAACCACTGA
CgHIFαlike- qRT-R	CCAGCAGCAACAGGAATCCATTCA
CgHIFα- qRT-F	TCAGGATTACCAGGAGCCGACTAA
CgHIFα- qRT-R	GCAAGCGTTTACCACCAGAGAGG
CgHIFαlike-qRT-aF	TGGCTGAACGCGTGAATAAAGGATC
CgHIFαlike -qRT-aR	ACTTCTCTGCTCTTGTTAGTCTCCG
CgHIFαlike -qRT-bF	GTGAATGATTGTAAGTTGAACAGGT
CgHIFαlike -qRT-bR	CTCGACTTCTCTGCTCTTGT
CgHIFαlike -qRT-cF	GGTTCTTTAAGCATTATTGAGAGAC
CgHIFαlike -qRT-cR	CTCGACTTCTCTGCTCTTG
CgHIFαlike -qRT-dF	CAGTACGGTGTCATAGTAGCGGAGT
CgHIFαlike -qRT-dR	GGAGATTTTTCCTCAGTGGTTGGCT
CgARNT- qRT-F	TCGGACGAGAACTCAAGCACAC
CgARNT- qRT-R	GTAACCAAGAACGGCTGTCACTCT
EF- qRT-F	ACCACCCTGGTGAGATCAAG
EF- qRT-R	ACGACGATCGCATTTCTCTT
Prokaryotic expression vector	
pET-CgHIFαlike-F	TATCGGATCCGAATTCATGTCAGTGTACAAAAAAATAAAGA
pET-CgHIFαlike-R	GACGGAGCTCGAATTCATTACTGGTGTTGTAAATTATGGT
pET-CgHIFα-F	TATCGGATCCGAATTCATGAGCTCTAATACGAAAAGAAGGA
pET-CgHIFα-R	GACGGAGCTCGAATTCCAGCATGGGAACTTGCTGGCTA
pGAD-CgHIFαlike-F	GGAGGCCAGTGAATTCATGTCAGTGTACAAAAAAATAAAGAT
pGAD-CgHIFαlike-R	CGAGCTCGATGGATCCTCAAAATGGATAATGACGCG
pGAD-bHLH/PAS -F	GGAGGCCAGTGAATTCATGTCAGTGTACAAAAAAATAAAGA
pGAD-bHLH/PAS -R	CGAGCTCGATGGATCCATTACTGGTGTTGTAAATTATGGT
pGAD-Del -F	GGAGGCCAGTGAATTCTACATCATCAGTGAGGAGGAAGG
pGAD-Del -R	CGAGCTCGATGGATCC TCTTTGTTTCTCCATTGCACGG
pGBK-CgARNT-F	CATGGAGGCCGAATTCATGGATTACTCTGATCTTCC
pGBK-CgARNT-R	GCAGGTCGACGGATCCTGGTTGTTCGCTTGGCATT
Eukaryotic expression vector	
pcDNA- CgHIFαlike-F	GTGGCGGCCGCTCGAGATGTCAGTGTACAAAAAAATAAAGAT
pcDNA- CgHIFαlike-R	GCCCTCTAGACTCGAGTGGATAATGACGCGTCTTCT
pcDNA- CgHIFα-F	GTGGCGGCCGCTCGAGATGAGCTCTAATACGAAAAGAAGGA
pcDNA- CgHIFα-R	GCCCTCTAGACTCGAGCATCAGCTCTGAGTCAGGT

For phylogenic analysis, full sets of bHLH-PAS sequences in the represented species were obtained from GenBank (NCBI; http://www.ncbi.nlm.nih.gov/genbank/) database; all the identified sequences are shown in S1 Table. Multiple alignments were performed using ClustalX. Phylogenetic analysis was conducted by neighbor-joining (NJ) method using MEGA5.0 software (http://www.megasoftware.net); the reliability of the estimated tree was evaluated by bootstrapped 1000 replicates.

### Semi-quantitative Real-time Polymerase Chain Reaction

Total RNA was extracted from each sample using 1.5mL TRIzol reagent (Invitrogen, Carlsbad, CA, USA). The first-strand cDNA was reverse transcribed from 1μg of the total RNA using PrimeScript RT reagent kit with gDNA Eraser (TaKaRa, Shiga, Japan), following the manufacturer’s instructions. Then semi-quantitative PCR was performed in an ABI 7500 Fast Real-Time PCR System (Applied Biosystems, Foster City, CA, USA) with SYBR Green PCR Master Mix (TaKaRa, Shiga, Japan). The primers are shown in Table 1. The cycling conditions used were as follows: 95°C for 30 s, followed by 40 cycles of 95°C for 5 s and 60°C for 30 s. At the end of the cycling stage, a melt curve analysis was performed to confirm that only one PCR product was amplified. Elongation factor (EF) was selected as the internal control, as described by Du et al. (2013) and Giannetto et al. (2015) [51, 53]; its expression was observed to be stable in our experiments. The data were then analyzed using the 2^-ΔΔCt^ method to estimate the relative expression level of the target genes.

### Plasmid Construction

The full-length *CgHIFα-like* cDNA was amplified by PCR using the thermostable DNA polymerase from *Thermococcus kodakaraensis* (KOD plus DNA polymerase; Toyobo, Japan). The amplified DNA was inserted into the mammalian expression vector pcDNA3.1/Myc-His (+) (Clontech, Mountain View, CA, USA), forming the Myc-tagged pcDNA-CgHIFαlike. The vector was linearized by XhoI digestion (New England Biolabs, Ipswich, MA, USA) and the amplified *CgHIFα-like* ORF was inserted into the vector using the In-Fusion HD Cloning Kit (Clontech), according to the manufacturer’s instructions. All the primers used are listed in Table 1. All the generated constructs were verified by DNA sequencing.

### Cell Culture, Transient Transfection, and Transcriptional Analysis

Human embryonic kidney (HEK) 293T cells (ATCC, Manassas, USA) were cultured in Dulbecco’s modified Eagle’s medium (DMEM) supplemented with 10% fetal bovine serum (FBS) and antibiotics (100 U/mL penicillin and 100 U/mL streptomycin) at 37°C and 5% CO_2_ in an incubator. Hypoxia treatment was administered in a hypoxia chamber with 1% O_2_.

For transfection, the cells were grown to 70–90% confluency; the intended plasmid DNAs were transfected into the cells using Opti-MEM and Lipofectamine 3000 (Life Technologies, Carlsbad, CA, USA), following the manufacturer’s protocol.

To determine the transcriptional activity of CgHIFα-like, each of the pcDNA-CgHIFαlike, pcDNA-CgHIFα, and pcDNA3.1 plasmids were transfected into HEK293 cells containing a wild-type p2.1 reporter gene or the control p2.4. p2.1 is a well-established HRE reporter gene, kindly provided by Professor Cunming Duan (University of Michigan, U.S.A.) and p2.4 was engineered by site-directed mutagenesis of all the HREs in p2.1, as described by Semenza et al. (1996) [54]. Additionally, pRL-SV40, a *Renilla luciferase* reporter plasmid, was cotransfected as a control for determining the transfection efficiency. The luciferase activity was measured by a dual-luciferase reporter assay system (Promega) in a Varioskan Flash multimode reader (Thermo Scientific, Waltham, MA, USA), following the manufacturer’s instructions. The results are reported as fold increases over the levels in the control group.

### Immunoblot analysis

HEK293T cells were cultured in 6-well plates and transfected with the target plasmids (2.5 μg per well), pcDNA-CgHIFαlike, pcDNA-CgHIFα, and pcDNA3.1vector, under normoxia and hypoxia. Cells were washed once with PBS, 24 h after the transfection, and lysed in RIPA buffer in the presence of protease and phosphatase inhibitors. Western blotting was performed with monoclonal anti-myc antibody (Roche, Penzberg, Germany) using Western Lightning Plus-ECL (PerkinElmer, Waltham, MA, USA) as the substrate. Briefly, the protein samples were separated by 10% SDS-PAGE and transferred onto a PVDF membrane. The membranes were blocked in 5% skimmed milk for 1 h and incubated with monoclonal anti-myc antibody (Roche, Penzberg, Germany) for 2 h. Subsequently, the membranes were washed thrice (5 min for each wash) with Tris-buffered saline (TBS) containing Tween-20 and incubated with the secondary antibody (horseradish peroxidase-conjugated goat anti-mouse IgG (Roche) for 1 h. Finally, after another round of three washings, the bands on the membranes were visualized by enhanced chemical luminescence. Mouse anti-β-actin IgG (ABclonal Technology, Cambridge, MA, USA) was used as a control.

### Electrophoretic Mobility Shift Assay

Prokaryotic expression vector pET30a (Novagen, George Town, KY, USA), containing the cDNA of interest, was used for the expression of recombinant proteins in *Escherichia coli* BL21 (DE3) (TransGen Biotech, China). Briefly, the DNA containing the bHLH-PAS domain of CgHIFα-like (aa1-385) or CgHIF-α (aa1-356) proteins was amplified and inserted into the pET30a plasmid and transformed into BL21 (DE3). After an overnight culture in liquid broth containing kanamycin (50 μg/mL), the transformed *E*. *coli* were added to fresh medium and grown to OD_600 nm_ = 0.5–0.6. IPTG was then added to the culture to a final concentration of 0.5 mM and incubated for an additional 12 h to express the target protein. Finally, the soluble recombinant protein was extracted from the supernatant of the *E*. *coli* lysate by ultra-sonication and stored at -80°C for further use. The concentration of recombinant proteins was determined by a BCA Protein Assay kit (Pierce Biotechnology, Rockford, IL, USA) and they were visualized on 12% SDS-polyacrylamide gels by staining with Coomassie brilliant blue R250.

Electrophoretic mobility shift assay (EMSA) was performed using a LightShift Chemiluminescent EMSA kit (Pierce, Rockford, USA), following the manufacturer’s instructions. Briefly, the proteins were obtained from the prokaryotic expression system as described above. The biotin end-labeled DNA probe (20 fmol) was then incubated with the target proteins (1 μg) in 20 μL binding buffer (pH 7.5) containing 10 mM Tris, 50 mM KCl, 1 mM dithiothreitol (DTT), and 50 ng poly (dI-dC). Binding reactions were carried out for 20 min at room temperature. Because no oyster HIFα consensus probe was available, the following biotin-labeled double-stranded oligonucleotide of human erythropoietin gene HIF-1α binding sites was used as a probe (sequence of the sense strand: Ew, 5′-GCCCTACGTGCTGTCTCA-3′). For competition experiments, a 200-fold molar excess of the cold probe (unlabeled annealed Ew,) or mutant probe was added to the binding reaction before addition of the labeled probe; the mutant oligonucleotide sequence used was as follows (the mutation is underlined): Em, 5′-GCCCTAAAAGCTGTCTCA-3′. For supershift analysis, a monoclonal anti-His antibody (ABclonal Technology, Cambridge, MA, USA) or the preimmune serum was added to the EMSA reaction mixture and incubated for an additional 20 min on ice before electrophoresis.

### Yeast Two-hybrid Assay

We isolated and cloned the cDNA sequence of CgARNT gene from the Pacific oyster *C*. *gigas*, as described above (S1 Fig). Yeast two-hybrid assay was performed with the Matchmaker™ Gold Yeast Two-Hybrid System (Clontech) according to the manufacturer’s protocol. Briefly, DNA encoding the full-length or the truncated versions (with or without the bHLH-PAS domain) of CgHIFα-like and CgARNT were amplified by PCR and subcloned into pGAD-T7 (pGAD-CgHIFαlike/bHLH-PAS/Del) and pGBK-T7 (pGBK-CgARNT) vectors and transformed into *Saccharomyces cerevisiae* strains Y187 and Y2H Gold (Clontech), respectively. Positive yeast strains were hybridized in 2× YPDA media at 30°C for 20–24 h and then the cotransformed yeasts were spread on SD/–Trp–Leu and SD/–Ade–His–Leu–Trp media (Clontech), supplemented with α-Xgal (Clontech) and Aureobasidin A (AbA, Clontech), for 3–5 days at 30°C. The successful interactions were determined by the presence of blue cells on the SD/–Ade–His–Leu–Trp media.

### Statistical Analysis

The values obtained in all the experiments are shown as mean ± SD. One-way analysis of variance (ANOVA) was used to test the differences in the values among the tissues used whereas Student’s *t*-test was used to analyze the differences between normoxia and hypoxia by using SPSS (v. 13.0; Chicago, IL, USA).

## Results

### Characterization of *CgHIFα-like*, a Novel Member of the Oyster bHLH-PAS Gene Family

In the present study, we identified and cloned the full-length cDNA of *CgHIFα-like* gene from the Pacific oyster *C*. *gigas*. The open reading frame (ORF) of *CgHIFα-like* was 2667 bp in length, encoding a protein of 888 amino acids with a predicted molecular weight of 98.4 kDa and pI of 6.37. Structural analysis demonstrated that CgHIFα-like shared a similar structural feature with bHLH-PAS proteins, including a bHLH DNA binding domain (aa 38–91) and two PAS domains (aa 136–200 and aa281–354). Besides, the arrangement and spacing of the domains were also conserved among other members of the bHLH-PAS superfamily. In our database search for *C*. *gigas*, we identified eight members of the bHLH-PAS family.

**Fig 1:**
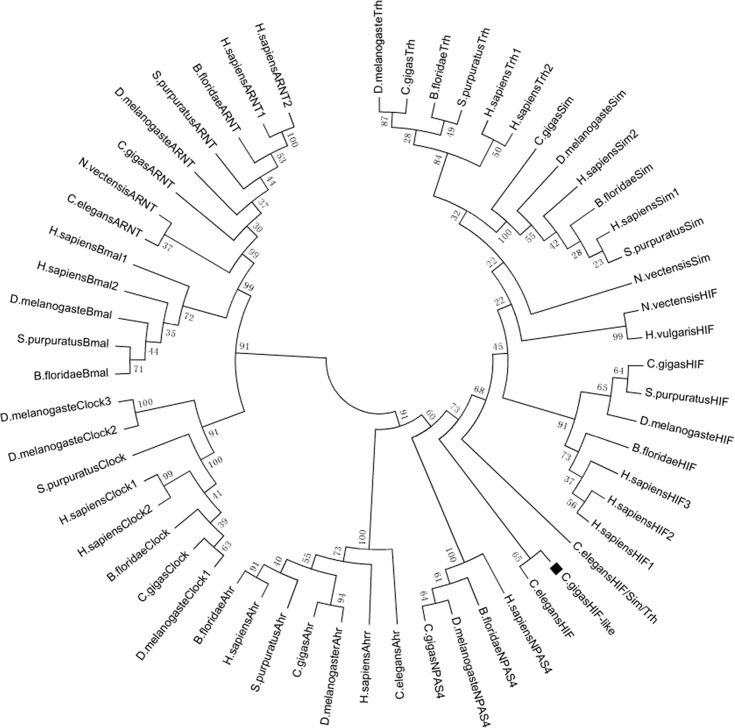
Phylogenetic analysis of bHLH-PAS system. Full-length protein sequences of bHLH-PAS family members in the representative species were aligned and analyzed by neighbor-joining (NJ) method with 1000 bootstrap using the MEGA5.0 software.

In the phylogenetic analysis, bHLH-PAS proteins were classified into two groups: Class I included the aryl hydrocarbon receptor (Ahr), neuronal PAS domain protein (NPAS), hypoxia inducible factors (HIF-1α/2α/3α), single-minded proteins (Sim1/2), and trachealess (Trh) protein; Class II consisted of the ubiquitous aryl hydrocarbon receptor nuclear translocator (ARNT) and the circadian rhythm related proteins, brain-muscle-ARNT-like transcription factor (Bmal) and circadian locomotor output cycles protein kaput (Clock; Fig 1). Similar to NvHIF and HvHIF, CgHIFα-like strongly clustered with the HIF, Sim, and Trh families; CgHIFα-like formed a well-supported subclade with CeHIF and is possibly a divergent member of the HIF family. Thus, the *CgHIFα-like* gene encodes a novel member of the bHLH-PAS protein family that is most closely related to the HIF protein responding to hypoxia.

### Identification of Multiple CgHIFα-like Isoforms and their Spatial Expression Patterns

**Fig 2:**
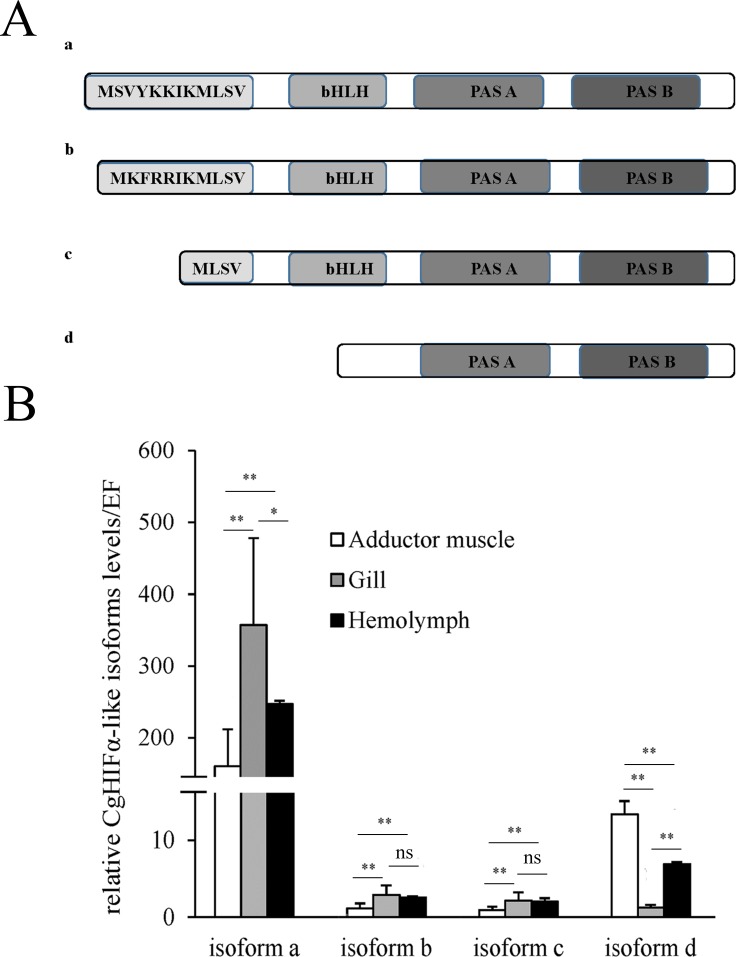
Characterization of the mRNA isoforms of CgHIFα-like and their expression pattern in different tissues. (A) Schematic diagram of the multiple CgHIFα-like isoforms identified by RACE. Characteristic domains are marked in black boxes. (B) Semi-quantitative PCR analysis of CgHIFα-like *isoforms a* to *d* transcripts in different tissues. The adductor muscle of *isoform a* was used as the reference sample and Elongation factor (EF) mRNA level was used as an internal control. A, adductor muscle; G, gill sample; H, hemolymph. Data are displayed as mean ± SD of independent triplicate experiments. Asterisks indicate significant differences at *P* < 0.05 * and *P* < 0.01** and ns, not significant.

We isolated four *CgHIFα-like* isoforms (*CgHIFα-like a*, *b*, *c*, and *d*) containing alternative 5′-untranslated regions and different predicted translational start sites (Fig 2A). Among the four CgHIFα-like isoforms, only the isoform a contained the complete amino acid (aa) sequence, whereas isoforms b, c, and d had a few aa deletions in the upstream region. Briefly, the *CgHIFα-like b* mRNA encoded a protein with a five N-terminal amino acid residue difference from the *CgHIFα-like a* mRNA-encoded protein, whereas *CgHIFα-like c* translated into a protein lacking seven amino acid residues upstream of bHLH domain compared to the long *CgHIFα-like a* isoform. *CgHIFα-like d* isoform, particularly, produced a short protein that lacked the bHLH domain. The sequences of all the four isoforms were deposited in GenBank (BankIt1868724 KU053515- KU053518), respectively.

We determined the expression patterns of the four *CgHIFα-like* isoforms in various tissues using the primers specific for each isoform (Fig 2B). The results showed that the transcripts of *isoforms a*, *b*, and *c* were most abundant in the gills, followed by adductor muscle and hemolymph, although no statistical significance was detected between the first two tissues both for *isoform b* and *c*). Interestingly, a marked difference in tissue distribution was observed in the mRNA of *isoform d*, which was 11- and 6-fold higher in the adductor muscleand hemolymph,respectively, than in gills.

### Hypoxic Regulation of Different CgHIFα-like Isoforms at mRNA and Protein Levels

**Fig 3:**
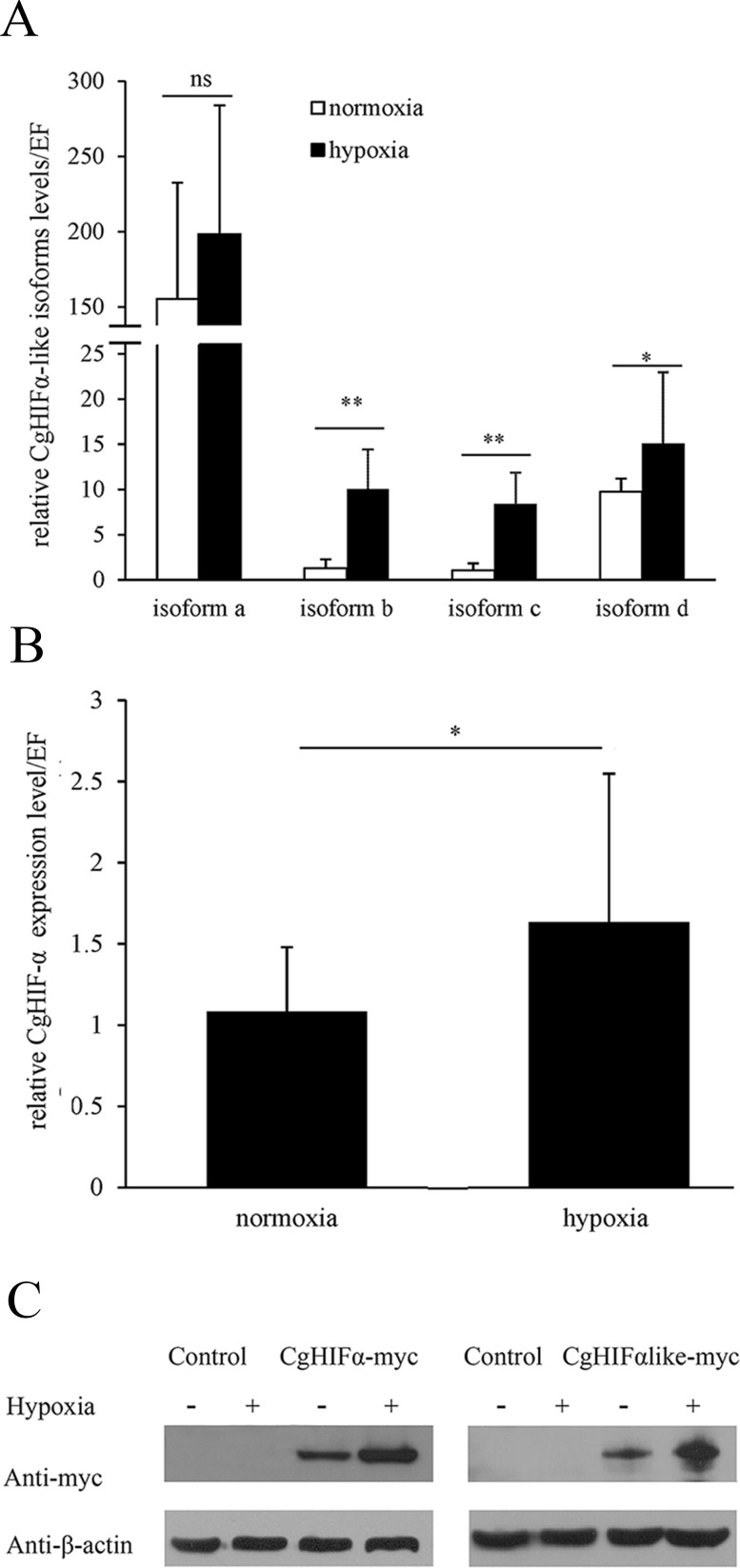
Effect of low oxygen on mRNA and protein levels of CgHIFα-like. (A-B) Semi-quantitative PCR analysis of CgHIFα-like *isoforms a* to *d* and *CgHIFα* transcripts under hypoxic treatment. Data are shown as the mean ± S.D (N = 3). Asterisks indicate significant differences at *P* < 0.05 * and *P* < 0.01** and ns, not significant; (C) Both the CgHIF-α and CgHIFα-like proteins were capable of responding to hypoxia. HEK 293T cells transfected with the indicated plasmids (CgHIF-α, CgHIFα-like) or empty vector were incubated under hypoxia (1% oxygen) or normoxia (ambient air); 24 h after the transfection, the cells were lysed and analyzed by immunoblotting with the corresponding antibodies. The protein levels were quantified by densitometry and normalized to β-actin levels. The experiments were conducted in triplicate.

We examined the mRNA expressions of various *CgHIFα-like* isoforms at low oxygen concentration by semi-quantitative PCR using a primer set targeting sequences specific to each isoforms (Fig 3A). The expression level of *CgHIFα* mRNA, which is a known hypoxia inducible factor gene in oysters, was used as a positive control (Fig 3B). Results showed that hypoxia treatment resulted in increases in all the isoforms, especially the *CgHIFα-like isoforms b* and *c*. *Isoforms b*, *c* and *d* mRNA levels were increased 7.8-, 8-, and 1.5-times after 72-h hypoxia exposure, respectively. Although the *isoform a* mRNA level was 1.3-fold higher than that of the control, the increase was not significant (P > 0.05).

We investigated the hypoxia response of CgHIFα-like at the protein level by western blotting using human HEK293T cells (Fig 3C). Because of the small difference in size among the isoforms, it was difficult to distinguish them at the protein level; we, therefore, decided to use the plasmid incorporating the longest isoform (i.e., isoform a) as the pcDNA-CgHIFlike–Myc expression plasmid. pcDNA-CgHIFα-Myc vector was used as a positive control. In the cells transfected with pcDNA-CgHIFαlike–Myc construct, the expression of CgHIFα-like was low under normoxia, and it increased 1.94-times under hypoxia. Similar results were observed in the CgHIFα-myc-transfected group, in which the hypoxia treatment resulted in a 1.87-fold increase in the CgHIFα levels. No band was detected in the empty vector group under either normoxic or hypoxic conditions. These data collectively suggested that CgHIFα-like expression was regulated in an oxygen-dependent manner.

### HRE-dependent Transcription Activity of CgHIFα-like

**Fig 4:**
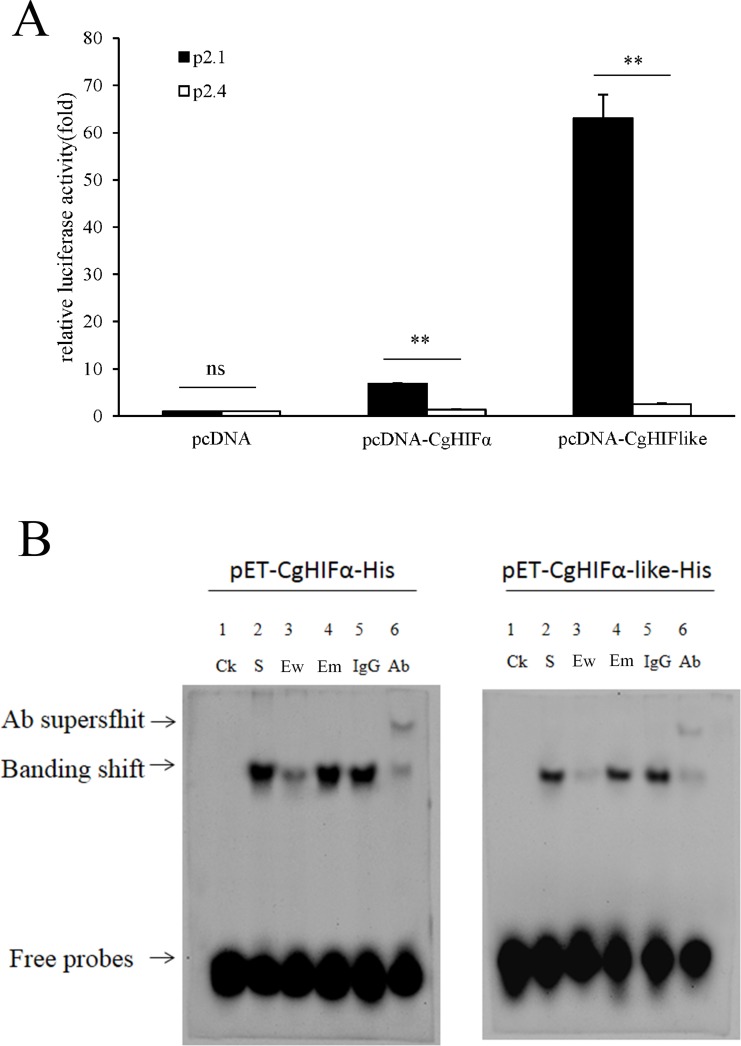
Transcription mechanism of CgHIFα-like. (A) Transactivation activities of CgHIFα-like. HEK 293T cells were transfected with the indicated pcDNA-CgHIFαlike, pcDNA-CgHIFα, or empty vector (control) plasmids together with p2.1 (solid bars) or the p2.4 (open bars) reporter plasmids; pRL-SV40 (*Renilla*) was cotransfected as an internal control. Transactivation activity is expressed as fold increase over the empty vector group. Data are displayed as mean ± SD of independent triplicate experiments. (B) Electrophoretic mobility shift assay for *in vitro* DNA binding of CgHIFα-like and CgHIFα. EMSA was performed using the recombinant proteins from the prokaryotic expression system. *Lane 1* (Ck) was a negative control, and contained only the biotin Ew probe; *Lane 2* (S) contained Ew probe as well as CgHIFαlike-His protein sample. For competition experiments, a 200 M excess of unlabeled human HRE Ew (*lane 3*) or mutant oligonucleotides Em (*lane 4*) of erythropoietin gene was used. For supershift experiment, binding reaction mixtures were incubated with a monoclonal anti-His antibody Ab (*lane 6*) or the preimmune serum IgG (*lane 5*).

The transcriptional activity of CgHIFα-like was determined using the wild-type p2.1 and the mutant p2.4 luciferase reporter plasmids in HEK 293T cells. As shown in Fig 4A, when co-transfected with recombinant CgHIFα-like vector, the transcription of reporter gene p2.1 was 25-fold higher than that of the p2.4 control reporter. Similarly, co-expression of CgHIFα with p2.1 caused a 5-fold increase compared to that observed with p2.4. However, in the pcDNA3.1-Myc group, no significant difference in the expression of the reporter gene was detected between the p2.1 and p2.4 co-transfection groups. These results suggested that CgHIFα-like was capable of specifically activating the HRE-dependent gene expression.

Electrophoretic mobility shift assay was performed to directly confirm the DNA binding of CgHIFα-like protein (Fig 4B). Similar to the positive control (CgHIF-α), when the recombinant CgHIFαlike-His protein was incubated with the labeled double-stranded oligonucleotide Ew, a DNA-binding complex was detected (Fig 4B, *lane 2*). The addition of 200-fold molar excess of unlabeled Ew attenuated or even eliminated the detection of protein-DNA binding activities (*lane 3*). In contrast, Em, containing the 3-bp substitution in Ew, failed to compete with the probe for binding of the proteins (*lane 4*). To further prove the specificity of DNA binding, we performed supershift assays. It demonstrated that the addition of monoclonal anti-His antibody against recombinant CgHIFα-like or CgHIFα resulted in a supershift band with reduced mobility (*lane 6*). In contrast, when the respective preimmune serum was added to the binding reaction, the mobility of the protein-probe complex was unaffected (*lane5*). These results demonstrate that CgHIFα-like could bind to the HRE sequence directly *in vitro*.

### CgHIFα-like Heterodimerizes with CgARNT

To ascertain whether CgHIFα-like dimerized with CgARNT and which CgHIFα-like domain was involved in the binding, we conducted a yeast two-hybrid assay to investigate the interaction of various CgHIFα-like deletion mutants with CgARNT. Before the experiment, we first identified CgARNT from *C*. *gigas* (S1 Fig). The *CgARNT* cDNA (3407 bp), contained start and stop codons at positions 749 and 2845, respectively; the predicted protein contained 698 residues and had a molecular weight of 76.97 kDa and a pI of 6.18. CgARNT shared only 42.7% sequence identity with the human ARNT, whereas the bHLH (positions 87–140) and two PAS (positions 159–230 and 362–413) regions appeared to be relatively well conserved (S2 Fig). Semi-quantitative PCR analysis showed that *CgARNT* mRNA level was lower in the adductor muscle than in the gills and hemolymph under normoxia, and no statistically significant changes in the response to hypoxia were detected in all the tissues studied. We constructed the pGBK-CgARNT recombinant protein and confirmed its interaction with both the full-length of CgHIFα-like and its bHLH/PAS domain; the deletion of bHLH/PAS domain abrogated the binding with CgARNT. It implied the formation of a heterodimer between CgHIFα-like and CgARNT, and suggested that the complex formation was dependent on the bHLH-PAS domain.

## Discussion

In the present study, we first identified and characterized a new bHLH-PAS gene family member named *HIFα-like* gene from the Pacific oyster. *CgHIFα-like* gene contains four isoforms that differ in their 5′-untranslated regions, and its expression could be induced by hypoxia at both the mRNA and protein levels. Besides, it has the ability to dimerize with CgARNT and activate the transcription of reporter gene by binding to a core hypoxia response element (HRE). Among all the invertebrates, this is the first report describing a novel bHLH-PAS family member responding to hypoxia besides HIFα, reported in the Pacific oyster.

Firstly, we cloned CgHIFα-like protein from *C*. *gigas*. The CgHIFα-like protein had conserved bHLH, PAS-A, and PAS-B domains, which indicates that it is a member of the bHLH-PAS family. To distinguish which sub-family it belonged to, we constructed a phylogenetic tree of all the eight members of bHLH-PAS proteins from *C*. *gigas*, together with other bHLH-PAS members from representative vertebrates and invertebrates. The result demonstrated that the bHLH-PAS superfamily was clearly classified into two groups, the Class I and Class II proteins. The best-characterized Class II bHLH/PAS factor is the ubiquitously present ARNT, which is a general partner of bHLH-PAS proteins; other members of class II include the circadian rhythm related proteins Bmal and Clock. The Class I proteins, including Ahr, NPAS, HIFα and Sim, must dimerize with a Class II bHLH-PAS factor to form active transcription factor complexes. In the Class I proteins, a small proportion of members were observed to be associated with more than one subfamily—for example, *N*. *vectensis* Sim, *N*. *vectensis* HIF, and *Hydra vulgaris* HIF were outgroups of Sim and Trh, whereas *C*. *elegans* HIF and CgHIFα-like proteins behaved as outgroups of the HIF, Sim, and Trh subfamilies. This phenomenon is common in the proteins of other species like *Homo sapiens* HIF2α, *Lottia gigantea* Lg60, and *Amphimedon queenslandica* Amp14; all of these tend to be external to the HIF/Sim/Trh families [55]. Besides, Woods and Whitelaw (2002) demonstrated that Sim1 protein, similar to HIF, has the ability to bind HRE and affect the activation of transcription [56]. Thus, there might be no clear boundaries among Sim/Trh/HIF families in some species. The close relation of CgHIFα-like with CeHIF indicates that it is possibly a novel member of the HIF family. However, unlike the known CgHIFα proteins, structural analysis showed that CgHIFα-like did not contain the typical LXXLAP motif (where *X* indicates any amino acid and *P* indicates the hydroxyl acceptor proline) in the ODD domain, which is related to the stabilization of HIF-α protein, although we found two potential proline residues in the corresponding regions. This phenomenon is widely observed in many invertebrate species; the LXXLAP motif was totally lost in the HIF-α ODD domain of *T*. *adhaerens* [39]; in *D*. *melanogaster* and *C*. *elegans*, there was a significant variation in the structure compared to the classical LXXLAP motif [42, 47]. It suggests that the ODD domain of HIF might have experienced considerable variation during early metazoan evolution. Besides, mutagenesis studies of Huang et al. (2002) also demonstrated that the two leucine residues in the LXXLAP motif were not essential for proline hydroxylation, and proline itself was absolutely necessary for PHD recognition [28]. Thus, CgHIFα-like protein may have an atypical ODD domain lacking the LXXLAP motif; however, further experiments are needed to confirm this hypothesis.

We cloned the four CgHIFα-like isoforms (*CgHIFα-like isoform a*, *b*, *c*, and *d*) from the oyster. The four cDNA isoforms of *CgHIFα-like* had overlapping C-terminal cDNA sequences but contained different 5′-untranslated regions and had different predicted translation start sites. Furthermore, we determined the expression patterns of these CgHIFα-like isoforms in different tissues. The results indicated that mRNAs of CgHIFα-like isoforms were constitutively expressed in all the studied tissues. The mRNA levels of *CgHIFα-like isoforms a*, *b*, and *c* were highest in the gills, although the transcript levels in the gills and hemolymph did not reach statistical significace for the *isoforms b* and *c*. In contrast, the *isoform d* transcript was most abundant in the adductor muscle. These different tissue distributions of the four mRNA isoforms might have been caused by their different upstream regions, especially for the *isoform d*. The *isoform d* mRNA produced the shortest *CgHIFα-like* protein lacking the whole bHLH domain, which showed markedly different spatial expression patterns compared to the other isoforms. In mammals, it is common to see different HIFα isoforms with only minor differences in the amino-terminal amino acids, which could lead to quite different tissue distribution. For example, the mouse 1.1 mRNA isoform encodes HIFα protein that is only 12 amino acids shorter than the isoform 1.2, but they showed distinct tissue distribution [57], similar result has also been found in humans [58, 59]. In addition, Gao et al. (2014) have identified several alternatively spliced HIFα isoforms from amphioxus, but they had variations in the ODD domain rather than in the N-terminus [60]. However, no HIFα isoforms have been reported from invertebrates earlier.

We further observed the mRNA expression of the different CgHIFα-like isoforms under hypoxic conditions. The results revealed that the hypoxia treatment caused an increase in the expression of all the isoforms, although the change in the expression of *isoform a* was not significant. The different degree of hypoxia induction might somewhat be due to the influence of their tissue distribution. For example, although *CgHIFα-like isoform a* transcripts showed no significant increase in gills and adductor muscle, it was significantly induced in hemolymph after 72h of hypoxia exposure (data not shown). In mammalian systems, the primary mechanism that regulates HIF-α abundance occurs at the post-translational level, whereas, recent studies have increasingly revealed the transcriptional regulation of HIF-α gene in many species. In vertebrates, HIF-α response to hypoxia is often transient and observed only during the onset of hypoxic conditions [61, 62]. In the crustaceans, short-term hypoxia significantly suppressed the mRNA levels of the HIF-α homolog in the white shrimp *Litopenaeus vannamei* [63] and the Atlantic blue crab *C*. *sapidus* [64], whereas, in the grass shrimp *P*. *pugio* it was constitutively expressed under moderate (2.5 mg/L DO) and severe (1.5 mg/L DO) hypoxia [40]. In contrast, in the small abalone *H*. *diversicolor*, HIF-α was up-regulated under hypoxia (2.0 mg/L DO) [49]. In the Eastern oyster, transcripts of HIF-α were not significantly affected by prolonged anoxia and hypoxia in most tissues except the gills, where the mRNA levels of HIF-α increased after prolonged exposure to moderate hypoxia [50]. Overall, the transcriptional response of HIF-α homologs to oxygen deficiency appeared to be variable, depending on the level of hypoxia and the duration of exposure, and showed species-specific differences, which in turn might be related to the degree of their hypoxia tolerance. To date, however, the regulatory transcription mechanism is still unclear, because no HRE has been found on the promoter regions of oyster CgHIFα-like. It may depend on other factors induced by HIF, but further experiments are needed to confirm this hypothesis. Immunoblotting showed that hypoxia could markedly induce high levels of CgHIFα-like. When tested in cultured human cells, CgHIFα-like protein significantly accumulated under hypoxic conditions, which is consistent with the reports in mammals. However, unlike in the typical mammalian system [65, 66], CgHIFα-like protein also expressed under normoxia in HEK293T cells, probably because this was an overexpression system. Future studies will be needed to determine the *in vivo* protein stability of CgHIFα-like.

Furthermore, we proved that CgHIFα-like was capable of binding to the HRE and activating the HRE-reporter gene transcription. The wild-type p2.1 and mutant p2.4 hypoxia reporter vectors were co-transfected with CgHIFα-like into HEK293 T cells, respectively. The results revealed that the expression of p2.1 was 25-fold relative to that of the p2.4, implying the HRE-specific transactivation of CgHIFα-like. Besides, the EMSA results further suggested that CgHIFα-like could bind directly and specifically to the HRE. All these data definitely verified that CgHIFα-like mediated the trans-activation of the reporter gene in an HRE-dependent manner, like its vertebrate homologs. However, the lack of conserved asparagine residue in CgHIF-like suggests that it probably contains an atypical transactivation domain, distinct from that of the CTADs of other HIF-α. However, further work is needed to map the functional activation domain of this region.

**Fig 5:**
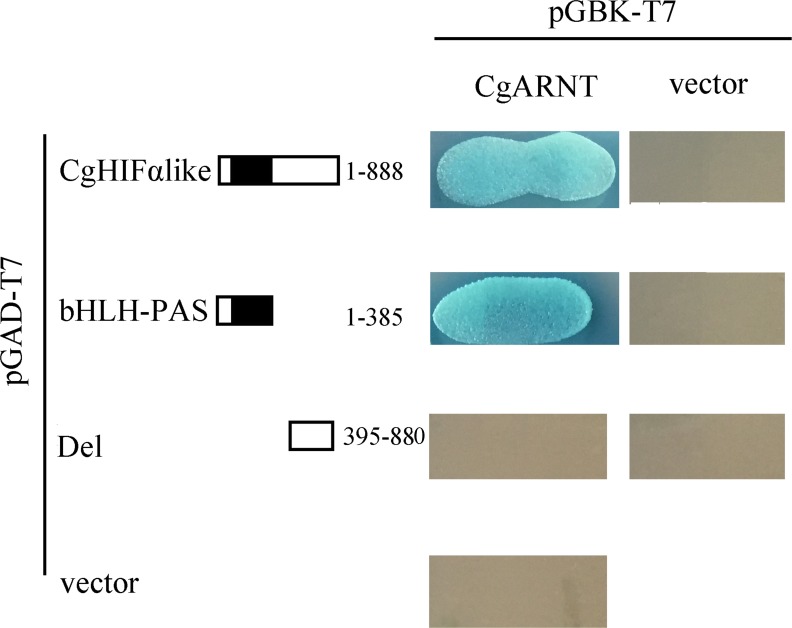
Yeast two-hybrid assay of interaction between CgHIFα-like and CgARNT. CgARNT was cotransformed with either full-length CgHIFα-like or its truncated versions; the positive yeast cells were then spread on the SD⁄–Trp–Leu and SD/–Ade–His–Leu–Trp media. All the experiments were performed in triplicate.

Finally, we cloned the partner (CgARNT) of CgHIFα-like from the Pacific oyster and confirmed that CgHIFα-like could dimerize with CgARNT. The substantial sequence conservation between CgHIFα-like and HIF-1α proteins from other species suggested that CgHIFα-like should interact with ARNT in much the same manner as the mammalian HIF-α. This prediction was reinforced by the existence of *CgARNT* gene in the Pacific oyster *C*. *gigas* [67]. To determine the interaction between CgHIFα-like and CgARNT, we performed the yeast two-hybrid analyses *in vitro* to estimate protein dimerization. As shown in Fig 5, all the cotransformed yeasts grew on the SD⁄–Trp–Leu medium. Besides, when the cotransformed yeasts containing the pGAD-CgHIFlike/pGBK-CgARNT vectors were spread on the SD/–Ade–His–Leu–Trp media for 3–5 days at 30°C, blue cells were observed on the media. This suggests that the two proteins physically interacted with each other *in vitro*. Thus, CgHIFα-like only interacted with CgARNT. In mammals, the bHLH-PAS domain is involved in dimerization and is essential for the interaction between HIFα and ARNT [68]. Besides, through the multiple sequence alignment, we found the bHLH/PAS domain of CgHIFα-like was highly conserved compared to the other species. To further confirm that only the conserved bHLH-PAS region of CgHIFα-like was responsible for CgHIFα-like/CgARNT heterodimerization, pGBK-CgARNT was cotransformed with differenttruncated CgHIFα-like proteins (DNA sequence with or without the bHLH-PAS domain); the results indicated that the bHLH-PAS domain was capable of binding with CgARNT, whereas the deletion mutant failed to interact with CgARNT. This demonstrated that the bHLH-PAS domain was necessary and sufficient for heterodimerization between CgHIFα-like and CgARNT. Together, these results demonstrate that CgHIFα-like heterodimerizes *in vitro* with CgARNT, and that this complex formation is mediated by the bHLH-PAS domain.

In summary, we cloned a novel bHLH-PAS transcription factor family member CgHIFα-like, and investigated its role under hypoxia stress in the Pacific oyster in addition to the traditional CgHIF-α. This is a first step towards understanding the special mechanism of HIF action in the mollusk species. These results suggest that the intertidal shellfish may have a more complex HIF metabolic regulation mechanism to adapt to the low oxygen environment. The findings of this study provide new insights into the unique mechanisms of hypoxia tolerance in mollusks.

## Supporting Information

S1 Fig cDNA sequence encoding oyster CgARNT and the predicted amino acid sequence. Nucleotides and amino acids are numbered on the left-hand side. The start (ATG) and stop (TAA) codons are underlined. The shaded areas indicate the conserved bHLH, PAS, and PAC domains, respectively.
(TIF)

S2 Fig Expression of CgARNT transcripts in different tissues. Oysters were kept in seawater with ambient O_2_ levels (normoxia) or were transferred to water with 20% of ambient O_2_ levels (hypoxia) for the indicated time. Elongation factor (EF) primers were used as internal control primers and the adductor muscle sample was used as the reference. Values are displayed as the mean ± SD (N = 3). A, adductor muscle; G, gill sample; H, hemolymph. Comparisons were carried out as follows: 1) within each tissue type, hypoxia treatment group was compared to the respective control, *P < 0.05 and ns, not significant; 2) within each oxygen level, mRNA levels were compared among the different tissues; different lowercase letters indicate values that are significantly different among the tissues within each experimental condition (P < 0.05).
(TIF)

S1 Table The reference bHLH-PAS sequences. (DOCX)
