# Supplementary figures and images for: Characterization of CgHIFα-Like, a Novel bHLH-PAS Transcription Factor Family Member, and Its Role under Hypoxia Stress in the Pacific Oyster Crassostrea gigas
Source: PLoS One. 2016 Nov 4;11(11):e0166057. doi: 10.1371/journal.pone.0166057 (PMC5096685; doi:10.1371/journal.pone.0166057)

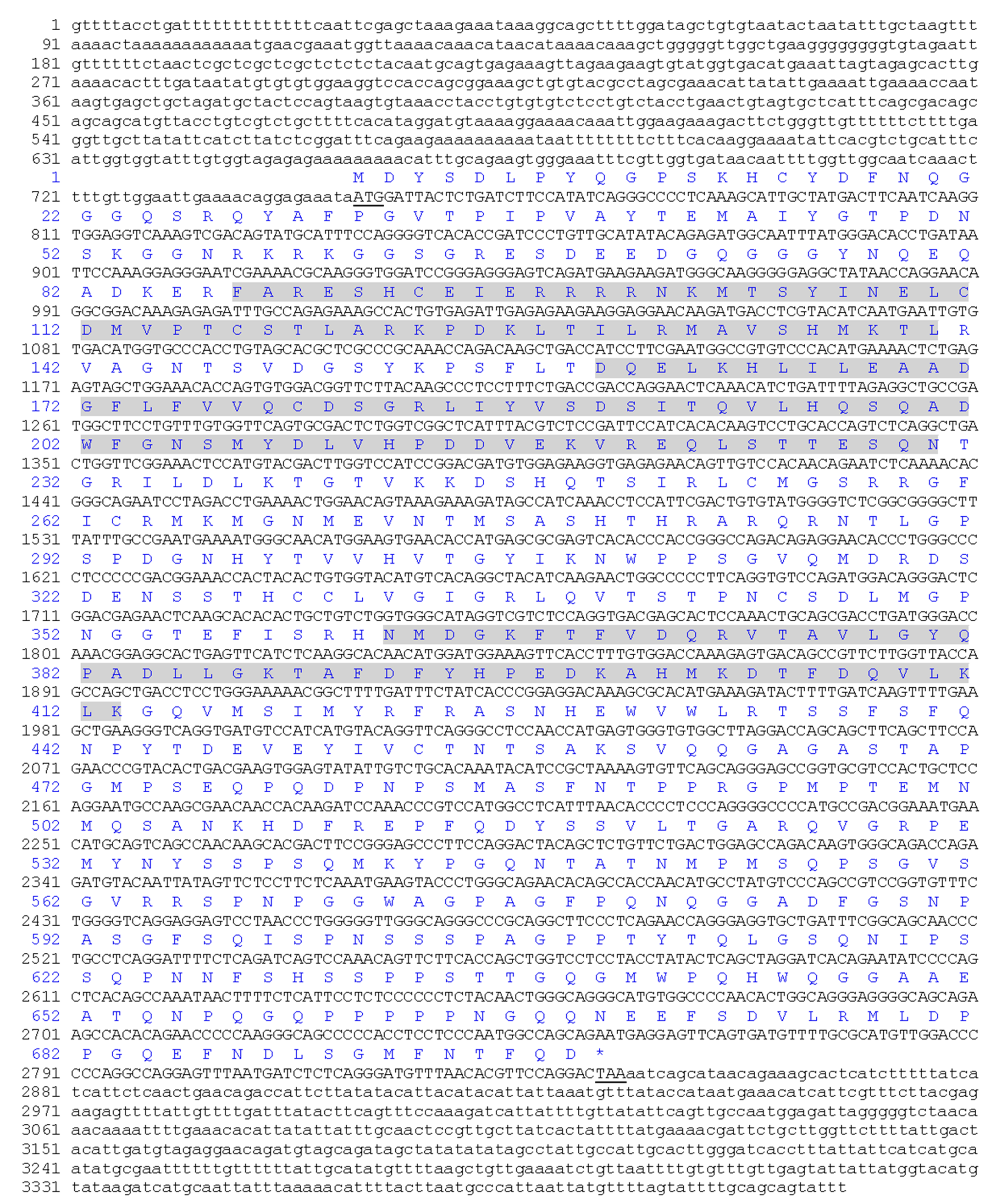

Supplement: S1 Fig — Nucleotides and amino acids are numbered on the left-hand side. The start (ATG) and stop (TAA) codons are underlined. The shaded areas indicate the conserved bHLH, PAS, and PAC domains, respectively. (TIF) [file pone.0166057.s001.tif]

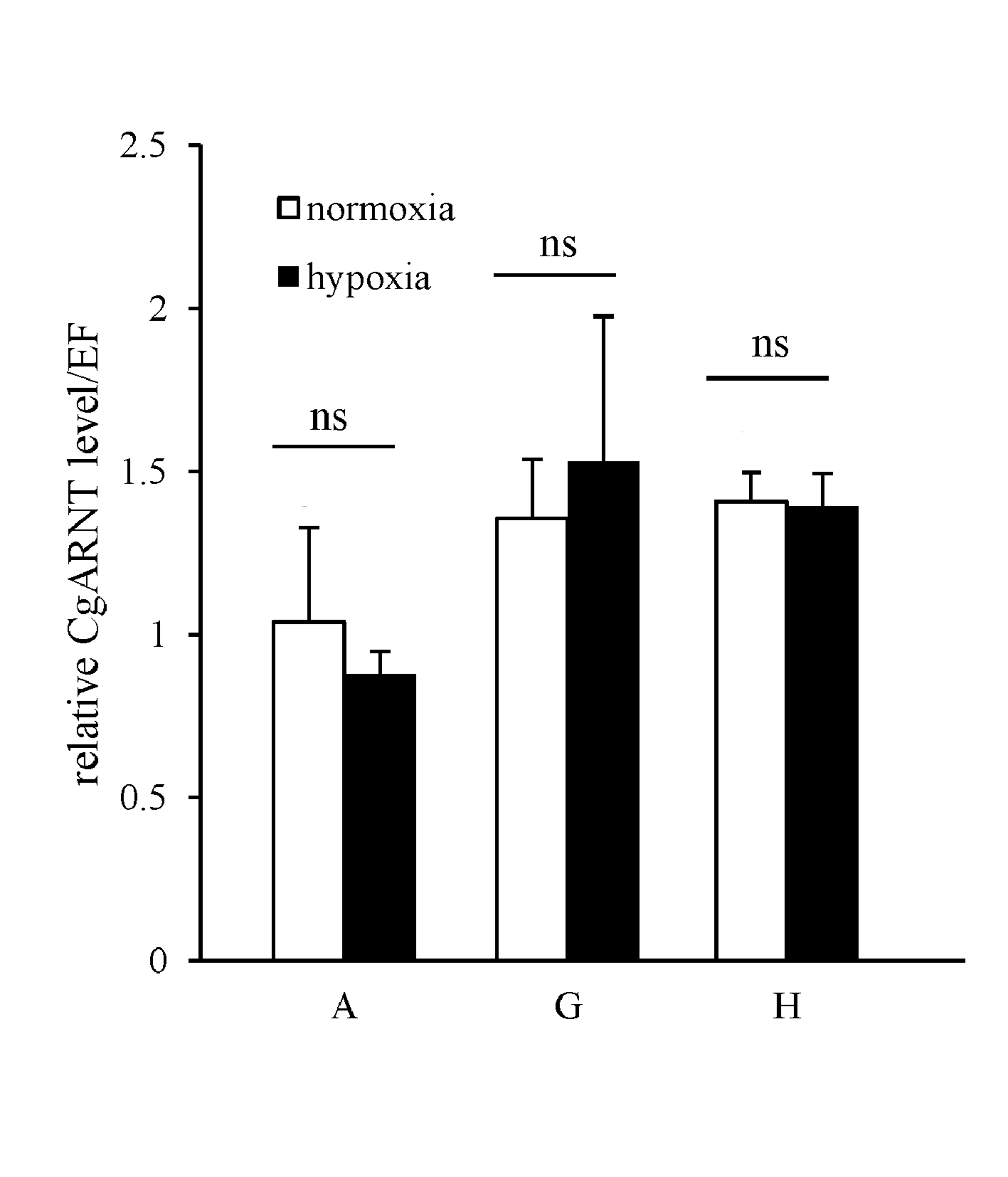

Supplement: S2 Fig — Oysters were kept in seawater with ambient O2 levels (normoxia) or were transferred to water with 20% of ambient O2 levels (hypoxia) for the indicated time. Elongation factor (EF) primers were used as internal control primers and the adductor muscle sample was used as the reference. Values are displayed as the mean ± SD (N = 3). A, adductor muscle; G, gill sample; H, hemolymph. Comparisons were carried out as follows: 1) within each tissue type, hypoxia treatment group was compared to the respective control, *P < 0.05 and ns, not significant; 2) within each oxygen level, mRNA levels were compared among the different tissues; different lowercase letters indicate values that are significantly different among the tissues within each experimental condition (P < 0.05). (TIF) [file pone.0166057.s002.tif]
