# Supplementary material for: Characterization of CgHIFα-Like, a Novel bHLH-PAS Transcription Factor Family Member, and Its Role under Hypoxia Stress in the Pacific Oyster Crassostrea gigas
Source: PLoS One. 2016 Nov 4;11(11):e0166057. doi: 10.1371/journal.pone.0166057 (PMC5096685; doi:10.1371/journal.pone.0166057)
Supplement: S1 Table — (DOCX) [file pone.0166057.s003.docx]

| species | name | Gene accession number |
| --- | --- | --- |
| *H.sapiens* |  |  |
|  | ARNT1 | NP_848513.1 |
|  | ARNT2 | NP_848514.1 |
|  | Bmal1 | AAL50340.1 |
|  | Bmal2 | NP_001025443.1 |
|  | HIF2 | NP_001521.1 |
|  | HIF1 | NP_001521.1 |
|  | Trh | NP_775182.1 |
|  | Trh2 | NP_002508.2 |
|  | Sim1 | NP_005059.2 |
|  | HIF3 | NP_690008.1 |
|  | Sim2 | NP_005060.1 |
|  | Clock2 | NP_002509.2 |
|  | Clock1 | NP_004889.1 |
|  | Ahr | NP_001612.1 |
|  | Ahrr | NP_065782.1 |
|  | NPAS4 | NP_849195.2 |
| *B.floridae* |  |  |
|  | Bmal | AGX25233.1 |
|  | ARNT | AGX25230.1 |
|  | Ahr | AGX25234.1 |
|  | Clock | AGX25232.1 |
|  | HIF | AGX25238.1 |
|  | Trh | AGX25237.1 |
|  | Sim | AGX25236.1 |
|  | NPAS4 | AGX25235.1 |
| *D.melanogaster* |  |  |
|  | Ahr | AAD09205.1 |
|  | NPAS4 | NP_001097936.1 |
|  | Trh | NP_523872.2 |
|  | Sim | NP_524340.2 |
|  | HIF | NP_001287599.1 |
|  | Clock1 | AAD10630.1 |
|  | Clock2 | AAC14350.1 |
|  | Clock3 | NP_511126.2 |
|  | ARNT | AAB88882.1 |
|  | Bmal | AAD10629.1 |
| *C.elegans* |  |  |
|  | Hif/Sim/Trh | NP_506391.2 |
|  | ARNT | NP_492687.1 |
|  | HIF | NP_001023893.1 |
|  | Ahr | NP_001021036.1 |
| *L.gigantea* |  |  |
|  | HIF | XP_009065767.1 |
|  | HIF/Sim/Trh | XP_009060317.1 |
| *T.castaneum* |  |  |
|  | Trh | EFA12054.1 |
|  | HIF | EFA04586.1 |
|  | Clock | NP_001106937.1 |
|  | Bmal | EFA01256.1 |
|  | Ahr | EEZ97710.1 |
| *S.purpuratus* |  |  |
|  | ARNT | XP_011679982.1 |
|  | Bmal | XP_011663797.1 |
|  | HIF | XP_783102.3 |
|  | Trh | XP_783845.2 |
|  | Sim | XP_782984.2 |
|  | Clock | XP_011672554.1 |
|  | Ahr | XP_011684106.1 |
| *N.vectensis* |  |  |
|  | HIF | XP_001637921.1 |
|  | Sim | XP_001635848.1 |
|  | Clock | XP_001639742.1 |
|  | Bmal | XP_001624731.1 |
|  | ARNT | XP_001624731.1 |
| *H.vulgaris* |  |  |
|  | HIF | XP_002167197.2 |
|  | ARNT | XP_012561289.1 |
